# Effect of 5:2 intermittent fasting diet versus daily calorie restriction eating on metabolic-associated fatty liver disease—a randomized controlled trial

**DOI:** 10.3389/fnut.2024.1439473

**Published:** 2024-08-20

**Authors:** Yuan-yuan Wang, Fang Tian, Xiao-lu Qian, Hui-min Ying, Zhen-feng Zhou

**Affiliations:** ^1^Department of Endocrinology, Xixi Hospital of Hangzhou, Hangzhou, Zhejiang, China; ^2^Department of Anesthesiology, Hangzhou Women’s Hospital (Hangzhou Maternity and Child Health Care Hospital, Hangzhou First People’s Hospital Qianjiang New City Campus, Zhejiang Chinese Medical University), Hangzhou, China

**Keywords:** calorie restriction, 5:2 diet, liver fat, daily calorie restriction, metabolic-associated fatty liver disease

## Abstract

**Objective:**

Both 5:2 IF diet (intermittent fasting) and daily caloric restriction eating had been suggested for management of MAFLD (Metabolic-Associated Fatty Liver Disease), this study aimed to evaluate the effects of 5:2 IF diet on body weight and metabolic parameters in adults with MAFLD, in comparison to daily caloric restriction eating.

**Methods:**

This single-center, double-blind, prospective, randomized controlled trial included 60 patients with MAFLD, who were administered either a 5:2 IF diet limited calories consumed for 2 days each week with no restrictions on the remaining 5 (Group 5:2 IF diet) or a daily calorie restriction eating (Group daily calorie restriction). Fibrotouch-B instrument assessment, ultrasound assessment of hepatic steatosis, anthropometric indices and body composition analysis, blood sample measurements were conducted during two distinct visits: initially on the day of study commencement (T1), and subsequently at the conclusion of the 12-week intervention period (T2).

**Results:**

In comparison to daily calorie restriction eating, the 5:2 IF diet significantly decreased the proportion of hepatic steatosis ≥moderate (29.6% vs. 59.3%, *p* = 0.028) and the degree of hepatic fibrosis *F* ≥ 2 (3.7% vs. 25.9%, *p* = 0.05), and fewer percentage of patients were diagnosed with fatty liver via upper abdominal ultrasound in the 5:2 intermittent fasting diet group (33.3% vs. 63.0%, *p* = 0.029). Additionally, the CAP (controlled attenuation parameter) and LSM (liver stiffness measurements) value were significantly lower in the 5:2 IF diet group (*p* < 0.05). No statistically significant differences were observed between the two groups in terms of weight, BMI (body mass index), WC (waist circumference), HC (hip circumference), and WHR (waist to hip ratio). Similarly, there were no significant differences in lipid profile, glycemic indices and adverse events (*p* > 0.05).

**Conclusion:**

In summary, although both 5:2 IF diet and daily caloric restriction eating achieved similar effect on body weight, liver enzymes, lipid profile and glycemic indices after 12 weeks treatment, 5:2 IF diet demonstrates better improvement in fibrosis and steatosis scores independently from weight regulation. Consequently, it is anticipated to emerge as a viable dietary modality for lifestyle intervention among patients diagnosed with MAFLD.

**Clinical trial registration:**

https://www.crd.york.ac.uk/PROSPERO, identifier ChiCTR2400080292.

## Introduction

MAFLD standed as the primary etiology behind chronic hepatic afflictions (1). Its development was tightly associated with unhealthy eating patterns rather than other factors that lead to obesity and metabolic changes (2). Within the management of MAFLD, dietary interventions retained considerable allure (3–5), with low-calorie regimens prominently advocated (5). However, it was difficult to maintain weight loss achieved by low-calorie diet (6), potentially stemming from provoking hunger sensations, thereby impeding sustained adherence (5).

Daily calorie restriction is a well-established primary weight-loss strategy for obese patients (7), and a protocol of time-restricted eating does not confer superior benefits in terms of body mass diminution, adiposity reduction, or mitigation of metabolic risk factors when compared to daily caloric restriction (7).

Currently, 5:2 IF (intermittent fasting) diet regimens had gained massive concern in patients with MAFLD (8, 9), driven by the heightened likelihood of patient acceptance and adherence to lifestyle modifications than taking medications daily (3). Adherence to the 5:2 IF diet has demonstrated beneficial effects compared to a standard diet among patients with non-alcoholic fatty liver disease (NAFLD), including reductions in weight, fat mass, and anthropometric markers of obesity, alongside improvements in hepatic steatosis, liver enzymes, triglycerides, and inflammatory biomarkers (8). However, in contrast to a daily calorie restriction diet, the results of improvement in visceral fat and some metabolic parameters by 5:2 IF diet was not consistent (10, 11). Furthermore, there remains a lack of sufficient evidence regarding the comparative impacts of the 5:2 IF diet versus daily caloric restriction on body weight and metabolic parameters in adults with MAFLD.

In light of these considerations, this study aimed to evaluate the effects of 5:2 IF diet on body weight and metabolic parameters in adults with MAFLD, in comparison to daily caloric restriction eating.

## Materials and methods

### Study participants

This single-center, prospective, randomized controlled trial was carried out from October 12, 2023 to March 28, 2024, and participants were recruited from the fatty liver obesity diagnosis and treatment center. This study was conducted according to the guidelines laid down in the Declaration of Helsinki and all procedures involving patients were approved by the Ethics Committee of Xixi Hospital of Hangzhou (IRB: 2021-045).

The inclusion criteria were as follows: (1) Age ≥ 18 years old; (2) Weight ≤ 155 kg; (3) There should be clinical evidence of fatty liver (liver biopsy, imaging, serum markers); (4) At least one of the three items of overweight/obesity, type 2 diabetes and metabolic disorder shall be combined. The definition of MAFLD is the presence of at least two metabolic abnormalities (12): (a) waist circumference increase: Asian population ≥90 cm (male) or 80 cm (female); (b) elevated blood pressure: ≥130/85 mmHg, or receiving antihypertensive medication treatment; (c) high triglycerides: ≥1.70 mmol/L, or receiving lipid-lowering medication treatment; (d) reduced high-density cholesterol (HDL-C):<1.0 mmol/L (male) or <1.3 mmol/L (female), or receiving medication treatment; (e) pre diabetes: fasting blood glucose 5.6–6.9 mmol/L, or blood glucose 7.8–11.0 mmol/L 2 h after meal, or glycated hemoglobin (HbA1c) 5.7–6.4%; (f) insulin resistance index (HOMA-IR) ≥ 2.5; (g) plasma hs-CRP (high-sensitivity C-reactive protein) >2 mg/L; (5) Signed an informed consent form, demonstrated willingness to participate in the experimental procedure, and voluntarily agreed to be a subject.

The exclusion criteria included: (1) Defined pathological conditions predisposing to hepatic steatosis, encompassing viral hepatitis, drug-induced hepatotoxicity, Wilson’s disease, and autoimmune hepatopathies; (2) Decompensated states of acute or chronic hepatic ailment; (3) Gastrointestinal disorders including diarrhea, diverticulosis, symptomatic irritable bowel syndrome, and inflammatory bowel disease; history of celiac disease; (4) Previous weight loss surgery or other abdominal surgeries; (5) Within 1 year, broad-spectrum antibiotic treatment with ≥3 courses of treatment; (6) A history of immunoglobulin deficiency within 2 months; (7) Pregnancy and patients with other underlying primary diseases and mental illnesses; (8) Patients with comorbidities such as tumors, severe cardiovascular and cerebrovascular diseases, and severe renal dysfunction are not suitable for selection as researchers in this study. No participant was exposed to vitamin E or steatogenic medications such as steroids.

### Randomization and blinding

An independent researcher conducted the randomization process utilizing computer-generated random-number software (Microsoft Excel, Redmond, WA, USA). Once the randomization lists were finalized, treatment allocation was determined by opening sealed, opaque envelopes, with this action taking place after the study subjects had given their consent to participate in the study.

Sixty patients were administered either a 5:2 IF diet limited calories consumed for 2 days each week with no restrictions on the remaining 5 (Group 5:2 IF diet) or a daily calorie restriction Eating (Group calorie restriction) prior to the commencement of the procedure.

Personnel involved in random allocation, diagnosis, and patient treatment were not engaged in data collection or follow-up procedures. The data collection and follow-up work is completed by an independent researcher who is not aware of the grouping situation, and a full-time statistician is responsible for statistical analysis of data, they do not participate in any other work.

#### Diet intervention

The intervention spanned a 12-week period for all participants enrolled in the randomized controlled trial (RCT). At the onset of the RCT, comprehensive dietary guidance was imparted to all subjects, emphasizing adherence to the prescribed dietary regimens. Regular monitoring throughout the study duration was facilitated through weekly telephone check-ins and monthly face-to-face interviews, wherein dietary recall information was collected via three 24-h recall assessments. Participants failing to adhere to the prescribed dietary regimen were excluded from further participation in the RCT.

#### The 5:2 IF diet

This dietary regimen entails customary eating habits for five consecutive days per week, maintaining an energy intake range of 1,400–1,600 kcal/d for females and 1,600–1,800 kcal/d for males (8). On the remaining 2 days, a regimen of light fasting is adopted, restricting daily energy intake to 600 kcal/day for females and 800 kcal/day for males.

#### Daily calorie restriction eating

Participants allocated to the daily calorie restriction group were instructed to adhere to prescribed caloric limits without temporal constraints. Throughout the 12-week trial duration, male participants were advised to adhere to a daily caloric intake ranging from 1,500 to 1,800 kcal, whereas female participants were instructed to consume between 1,200 and 1,500 kcal per day (7).

#### Dietary regimens for both groups

Both dietary regimens were designed to encompass a balanced macronutrient composition, with carbohydrates contributing 40–55% of total daily calories, protein accounting for 15–20%, and fat comprising 20–30% of total daily caloric intake. Notably, these dietary prescriptions mirrored recommendations outlined in prevailing dietary guidelines regarding macronutrient distribution (7).

Dietary counseling sessions were conducted by proficient health coaches, augmented by distribution of written informational booklets containing portion size recommendations and sample menus aligned with the prescribed dietary restrictions, consistent with contemporary dietary guidelines for macronutrient intake (7).

Detail of other treatment plans for MAFLD in this hospital was listed in Supplementary Appendix 1.

### Measurements and follow-up

#### Fibrotouch-B instrument assessment

The evaluation of hepatic steatosis and fibrosis was conducted employing the Fibrotouch-B instrument (FT/B-001-002, Wuxi Haskell Medical Technology Co., Ltd.). Measurements of liver fat alteration (CAP value) and liver elasticity (LSM value) were recorded (13). Evaluation of hepatic steatosis severity was delineated as follows: Normal: CAP value <240 dB/m; Mild: 240 dB/m ≤ CAP value <265 dB/m; Moderate: 265 dB/m ≤ CAP value <295 dB/m; Severe: CAP value ≥295 dB/m. The assessment of hepatic fibrosis severity was categorized as follows: F0 (absence of hepatic fibrosis): LSM value <7.3 KPA, F1: 7.3 KPA ≤ LSM value <9.7 KPA, F2: 9.7 KPA ≤ LSM value <12.4 KPA, F3: 12.4 KPA ≤ LSM value <17.5 KPA, F4: LSM value ≥17.5 KPA.

#### Ultrasound fatty liver

Ultrasound assessment of hepatic steatosis encompasses categorization into mild, moderate, and severe stages based primarily on parameters such as hepatic echotexture, parenchymal echogenicity, and attenuation of posterior echoes (8). Typical ultrasonographic features indicative of hepatic steatosis include heightened hepatic echogenicity with fine granularity, conspicuous visualization of intrahepatic vasculature, and increased hepatic volume. In mild hepatic steatosis, there is a discernible but moderate augmentation in liver volume accompanied by well-defined hepatic margins, while the hepatic parenchyma exhibits increased echogenicity. Moderate hepatic steatosis is characterized by liver boundaries that are moderately enlarged, with attenuated angles and parenchymal echoes exhibiting thickness and increased echogenicity. Severe hepatic steatosis is typified by markedly enlarged liver contours with blunted angles.

#### Anthropometric indices and body composition analysis

Body fat mass, body fat, muscle mass and visceral fat grade were evaluated through bioelectrical impedance analysis (BIA) (TANITA MC-780MA, Japan). The assessment took place following a 12-h fasting period. Participants were instructed to abstain from physical exertion 12 h before the test, empty their urinary bladder 30 min prior to measurements, and remove metallic objects immediately before the evaluation (8). For women of reproductive age, assessments were conducted outside the menstrual period.

#### Blood sample measurements

Blood samples (10 mL) were obtained from participants between 7 and 10 A.M., followed by centrifugation of the probes at 2,000*g* (RCF) at room temperature for 20 min. Serum biomarkers were analyzed both at the inception and conclusion of the RCT.

Liver enzymes, namely alanine transaminase (ALT), aspartate transaminase (AST), alongside lipid profile parameters including total cholesterol (TC), HDL-C, low-density cholesterol (LDL-C), and triglycerides (TG), were quantified employing standard methodologies recommended by Delta-dp diagnostic kits (AU5800, Beckman Coulter, Inc., USA). Inflammatory biomarkers of hs-CRP (Aristo, Shenzhen Guosai Biotechnology Co., Ltd., China) and serum insulin levels were evaluated via the ELISA method (ARCHIECT12000, Abbott Laboratories, USA). To assess insulin resistance, the homeostasis model assessment of HOMA-IR was calculated using the following formula: fasting insulin (μU/L) × fasting glucose (nmol/L)/22.5 (8). All participants underwent measurements of body weight, height, WC, HC, and WHR. Detailed measurement procedures are provided in Supplementary Appendix 1.

Evaluation of the mentioned parameters was conducted during two distinct visits: initially on the day of study commencement (T1), and subsequently at the conclusion of the 12-week intervention period (T2) (13).

#### Primary outcome

The proportion of hepatic steatosis ≥moderate was evaluated by Fibrotouch-B instrument at T2 (13).

#### Sample size

There are research reported (14) that compared to a normal diet, Mediterranean diet therapy can reduce the proportion of non-alcoholic fatty liver disease patients with fatty degeneration ≥ grade 2 from 93 to 48%. The main efficacy analysis aims to demonstrate that 5 + 2 light fasting treatment can also significantly reduce the proportion of non-alcoholic fatty liver disease patients with fatty degeneration ≥ grade 2 at 12 weeks after treatment, using a bilateral *X*^2^ test, *α* = 0.05, detection efficiency *β* = 90%. Each group requires 22 cases, assuming a dropout rate of 20%, each group requires 27 cases, for a total of 54 cases. Based on the main observation indicators, we used G-power software (G-Power version 3.1; The Institute for Experimental Psychology in Dusseldorf, Germany) to calculate the sample size.

### Statistical analysis

The data were analyzed using SPSS 20.0 (SPSS, Chicago, IL, USA) and measured as mean ± standard deviation and 95% confidence intervals (95% CI). The differences between groups were compared using the unpaired t-test, and the count data were compared by the *X*^2^ test or the Fisher exact probability method. Continuous variables with a nonnormal distribution were analyzed using a nonparametric test (Mann–Whitney U test). Paired t-tests were used for comparison before and after treatment, and Pearson analysis was used for correlation analysis. The comparison of repeated measurement data such as imaging examination and liver function examination between two groups was conducted using analysis of variance (covariance) of repeated measurement data. The two-sided *p*-value shows that the statistical significance is limited to *p* < 0.05.

## Results

### Baseline clinical parameters

**Figure 1:**
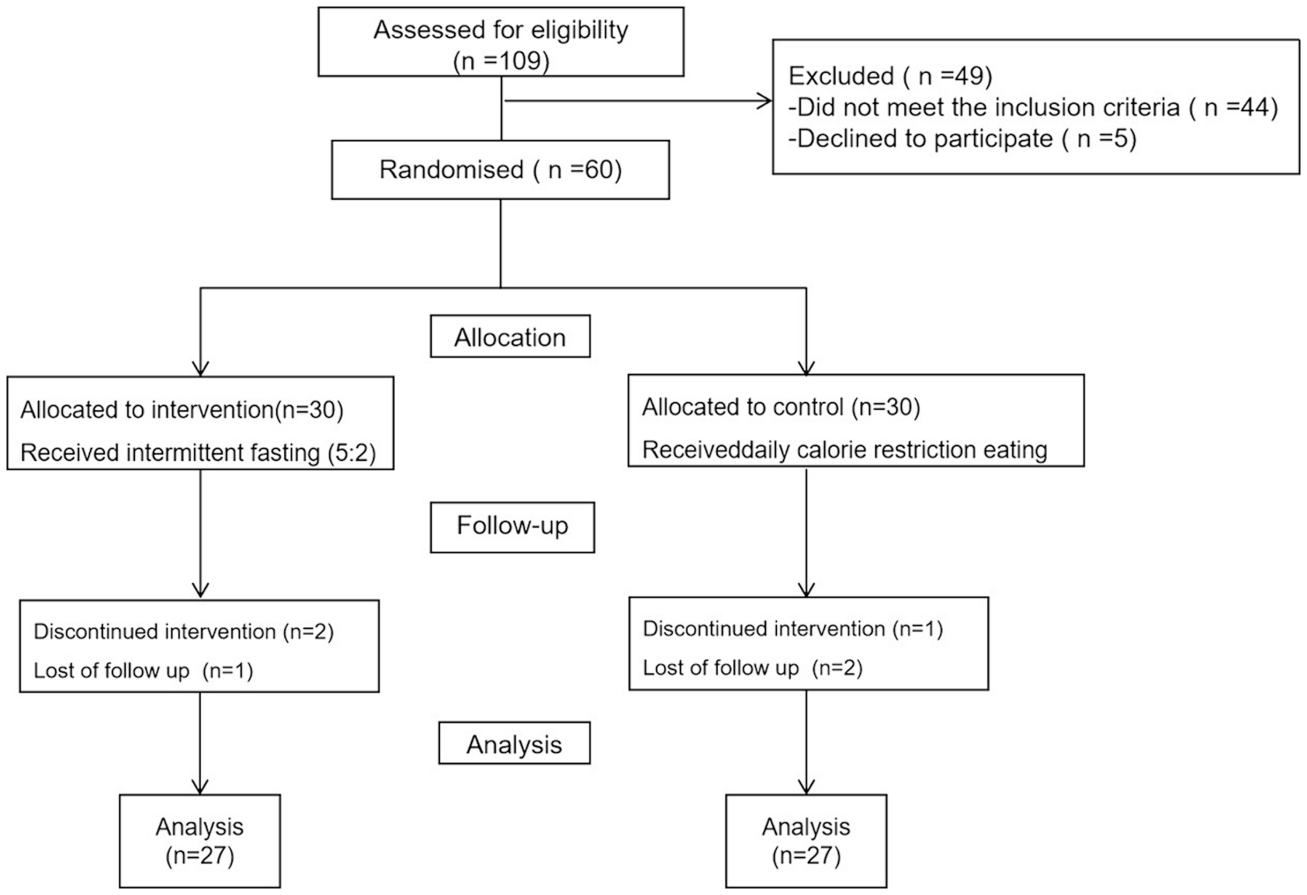
CONSORT flow diagram.

Initially, a total of 109 patients were enrolled in the study; however, 44 were excluded for not meeting the inclusion criteria, and five declined participation. Six participants who did not adhere to the prescribed dietary regimen were excluded from the study: two from the 5:2 IF diet group due to discontinued intervention, and one lost to follow-up; similarly, one participant from the calorie restriction group discontinued the intervention, and two were lost to follow-up. Consequently, the final analysis was conducted on a cohort of 54 patients, as illustrated in Figure 1. The mean age, weight, BMI, WC, HC, WHR of the patients were 31 ± 9 years, 93 ± 16 kg, 33 ± 5 kg/m^2^, 104 ± 10 cm, 111 ± 9 cm, and 0.93 ± 0.06%, respectively. No significant differences were observed between the groups concerning age, weight, BMI, WC, HC, WHR, anthropometric indices, body composition, hepatic parameters, lipid profile, and glycemic indices, however, in comparison to daily calorie restriction eating, total energy intake per day was significant lower in the 5:2 IF diet group (1,297 ± 469 vs. 1,524 ± 188, *p* < 0.001) (Table 1).

**Table 1:** Comparison of general data of patients between two groups.

Indicator	5:2 IF diet (*n* = 27)	Daily calorie restriction eating (*n* = 27)	*p* value
Age, year	32 ± 7	29 ± 10	0.150
Gender (female/male)	14/13	9/18	0.169
Height, cm	167 ± 8	170 ± 7	0.228
Weight, kg	92 ± 16	93 ± 15	0.873
BMI, kg/m^2^	33 ± 5	32 ± 5	0.555
Total energy intake/day, kcal	1,297 ± 469	1,524 ± 188	<0.001
**Hepatic steatosis** (*n*, %)			
Degree of hepatic steatosis			
Moderate/severe	22 (81.5%)	25 (92.6%)	0.420#
Degree of F0-1 of hepatic fibrosis	18 (66.7%)	15 (55.6%)	0.402
CAP, dB/m	310 ± 48	309 ± 39	0.950
LSM, kPa	7.0 ± 1.8	7.4 ± 2.1	0.441
Fatty liver by upper abdominal ultrasound (*n*, %)	27 (100%)	27 (100%)	>0.999
WC, cm	103 ± 11	104 ± 10	0.686
HC, cm	112 ± 9	111 ± 10	0.687
WHR, %	92 ± 7	94 ± 5	0.247
**Anthropometric indices and body composition**			
Body fat mass, kg	36 ± 10	34 ± 13	0.590
Body fat, %	34 ± 10	36 ± 10	0.547
Muscle mass, kg	59 ± 12	56 ± 10	0.258
Visceral fat grade	15 ± 5	15 ± 4	0.643
**Hepatic parameters**			
ALT, U/L	72 (60–91)	63 (30–120)	0.320
AST, U/L	44 ± 17	45 ± 28	0.834
**Lipid profile**			
TC, nmol/L	5.1 ± 0.9	5.1 ± 0.8	0.885
TG, nmol/L	1.7 (1.2–2.5)	1.9 (1.2–3.1)	0.494
HDL-C, nmol/L	1.2 ± 0.7	1.0 ± 0.2	0.242
LDL-C, nmol/L	2.6 ± 1.3	2.8 ± 0.8	0.444
**Glycemic indices**			
FBS, mmol/L	5.6 ± 0.7	5.5 ± 0.8	0.482
HbA1c, %	5.8 ± 0.5	5.8 ± 0.5	0.675
Fasting insulin, uIU/mL	13 ± 5	16 ± 8	0.129
HOMA-IR	3.3 (2.0–4.6)	3.1 (2.0–6.8)	0.505
**Inflammatory biomarkers**			
hs-CRP, mg/L	3.9 (2.1–5.7)	3.5 (1.3–5.3)	0.717
**Other**			
25(OH)D, ng/mL	20 ± 5	17 ± 6	0.149

#: Fisher’s exact probability; IF, intermittent fasting; BMI, body mass index; CAP, controlled attenuation parameter; LSM, liver stiffness measurements; WC, waist circumference; HC, hip circumference; WHR, waist to hip ratio; ALT, alanine aminotransferase; AST, aspartate aminotransferase; TC, total cholesterol; TG, riglycerides; HDL-C, high-density cholesterol; LDL-C, low-density cholesterol; FBS, fasting blood sugar; HbA1c, glycated hemoglobin; HOMA-IR, insulin resistance index; hs-CRP, high-sensitivity C-reactive protein; 25(OH)D, 25-hydroxyvitamin D.

### Effect on hepatic fibrosis and steatosis score

In comparison to daily calorie restriction eating, the 5:2IF diet significantly decreased the proportion of hepatic steatosis ≥moderate (29.6% vs. 59.3%, *p* = 0.028) and the degree of hepatic fibrosis *F* ≥ 2 (3.7% vs. 25.9%, *p* = 0.05). Fewer percentage of patients were diagnosed with fatty liver via upper abdominal ultrasound in the 5:2 IF diet group compared to the daily calorie restriction eating group (33.3% vs. 63.0%, *p* = 0.029). Additionally, the CAP and LSM value were significantly lower in the 5:2 intermittent fasting diet group than in the daily calorie restriction eating group (*p* < 0.05) (Table 2).

**Table 2:** Comparisons of the treatment effect between 5:2 IF diet and daily calorie restriction eating.

Clinical observation indicator	5:2 IF diet (*n* = 27)		*p*-value*	Daily calorie restriction eating (*n* = 27)		*p*-value*	*p*-value^&^
	Prior treatment	12 week post treatment		Prior treatment	12 week post treatment		
Weight, kg	92 ± 16	88 ± 17	0.407	93 ± 15	88 ± 15	0.249	0.942
BMI, kg/m^2^	33 ± 5	31 ± 5	0.223	32 ± 5	30 ± 5	0.179	0.445
**Hepatic steatosis** (*n*, %)							
Degree of hepatic steatosis							
Moderate/severe	22 (81.5%)	8 (29.6%)	<0.001	25 (92.6%)	16 (59.3%)	0.009#	0.028
Degree of F0-1 of hepatic fibrosis	18 (66.7%)	26 (96.3%)	0.011#	15 (55.6%)	20 (74.1%)	0.154	0.050#
CAP, dB/m	310 ± 48	235 ± 44	<0.001	309 ± 39	258 ± 37	0.008	0.045
LSM, kPa	7.0 ± 1.8	5.0 ± 1.4	<0.001	7.4 ± 2.1	6.1 ± 1.4	0.009	0.005
Fatty liver by upper abdominal ultrasound (*n*, %)	27 (100%)	9 (33.3%)	<0.001	27 (100%)	17 (63.0%)	0.001#	0.029
WC, cm	103 ± 11	95 ± 13	0.014	104 ± 10	97 ± 9	0.004	0.607
HC, cm	112 ± 9	108 ± 10	0.111	111 ± 10	106 ± 10	0.101	0.582
WHR, %	92 ± 7	88 ± 7	0.032	94 ± 5	91 ± 5	0.025	0.095
**Anthropometric indices and body composition**							
Body fat mass, kg	36 ± 10	30 ± 15	0.131	34 ± 13	29 ± 13	0.156	0.712
Body fat, %	34 ± 10	32 ± 10	0.293	36 ± 10	33 ± 11	0.307	0.567
Muscle mass, kg	59 ± 12	54 ± 13	0.149	56 ± 10	53 ± 9	0.333	0.732
Visceral fat grade	15 ± 5	12 ± 5	0.011	15 ± 4	12 ± 4	0.007	0.776
**Hepatic parameters**							
ALT, U/L	72 (60–91)	27 (17–35)	<0.001	63 (30–120)	24 (18–38)	0.001	0.979
AST, U/L	44 ± 17	29 ± 14	<0.001	45 ± 28	30 ± 18	0.024	0.768
**Lipid profile**							
TC, nmol/L	5.1 ± 0.9	4.8 ± 0.7	0.233	5.1 ± 0.8	4.6 ± 1.4	0.088	0.448
TG, nmol/L	1.7 (1.2–2.5)	1.3 (0.8–1.7)	0.049	1.9 (1.2–3.1)	1.6 (0.9–2.3)	0.082	0.337
HDL-C, nmol/L	1.2 ± 0.7	2.0 ± 1.0	<0.001	1.0 ± 0.2	1.0 ± 0.2	0.387	<0.001
LDL-C, nmol/L	2.6 ± 1.3	1.9 ± 1.1	0.032	2.8 ± 0.8	2.7 ± 0.6	0.378	0.003
**Glycemic indices**							
FBS, mmol/L	5.6 ± 0.7	5.3 ± 0.7	0.073	5.5 ± 0.8	4.9 ± 0.6	0.010	0.071
HbA1c, %	5.8 ± 0.5	5.6 ± 0.3	0.042	5.8 ± 0.5	5.4 ± 0.4	0.018	0.155
Fasting insulin, uIU/mL	13 ± 5	12 ± 11	0.534	16 ± 8	10 ± 6	0.005	0.547
HOMA-IR	3.3 (2.0–4.6)	2.0 (1.2–2.9)	0.021	3.1 (2.0–6.8)	1.8 (1.2–2.9)	0.002	0.710
**Inflammatory biomarkers**							
hs-CRP, mg/L	3.9 (2.1–5.7)	2.5 (1.3–4.6)	0.239	3.5 (1.3–5.3)	1.5 (0.8–3.5)	0.039	0.087
**Other**							
25(OH)D, ng/mL	20 ± 5	25 ± 4	<0.001	17 ± 6	24 ± 7	<0.001	0.256

#: Fisher’s exact probability; *: Comparisons of the treatment effect before and after treatment; &: Comparison of clinical observations after 12 W-treatment between the two groups. IF, intermittent fasting; BMI, body mass index; CAP, controlled attenuation parameter; LSM, liver stiffness measurements; WC, waist circumference; HC, hip circumference; WHR, waist to hip ratio; ALT, alanine aminotransferase; AST, aspartate aminotransferase; TC, total cholesterol; TG, riglycerides; HDL-C, high-density cholesterol; LDL-C, low-density cholesterol; FBS, fasting blood sugar; HbA1c, glycated hemoglobin; HOMA-IR, insulin resistance index; hs-CRP, high-sensitivity C-reactive protein; 25(OH)D, 25-hydroxyvitamin D.

### Effect on body weight, anthropometric indices and body composition

No statistically significant differences were observed between the two groups in terms of weight, BMI, WC, HC, and WHR. Similarly, there were no significant differences in anthropometric indices or body composition (*p* > 0.05) (Table 2).

Effect on hepatic parameters, lipid profile, glycemic indices and inflammatory biomarkers.

HDL-C levels was significantly higher and LDL-C significantly was lower in the 5:2 IF diet group compared to the daily calorie restriction eating group (*p* < 0.05). However, no significant differences were observed in ALT, AST, TC, TG, hs-CRP and glycemic indices (*p* > 0.05) (Table 2).

### Comparisons of the treatment effect before and after treatment

Table 2 presents a comparison of treatment effects before and after treatment in the two groups. At week 12 post-treatment, significant reductions were observed in the ratio of hepatic steatosis degree, CAP, LSM, the ratio of fatty liver diagnosed by upper abdominal ultrasound, WC, WHR, ALT, AST, visceral fat grade, 25 hydroxyvitamin D (25(OH)D), HbA1c, and HOMA-IR compared to baseline values (*p* < 0.05) in both groups. Additionally, a significant improved in the ratio of F0-1 hepatic fibrosis degree, TG, HDL-C and LDL-C were noted in the 5:2 IF diet group after 12-week treatment (*p* < 0.05), however, lower FBS, fasting insulin and hs-CRP were noted in the daily calorie restriction eating group (*p* < 0.05). There were no significant changes in BMI, body fat, body fat rate and muscle mass before and after treatment in either group (*p* > 0.05) (Table 2).

### Adverse events

Three subjects documented experiencing sensations of hunger and fatigue on fasting days, whereas five participants expressed various discomforts such as feeling cold, headaches, diminished energy levels, and sporadic dizziness within the group adhering to daily calorie restriction. It is noteworthy that no fatalities or severe adverse incidents were documented throughout the duration of the trial.

## Discussion

Our investigation revealed that both the 5:2 IF diet and daily calorie restriction regimens yielded comparable benefits in terms of liver enzymes, lipid profile, and glycemic indices following a 12-week intervention. However, the 5:2 IF diet exhibited superior amelioration in fibrosis and steatosis scores, along with specific markers of MAFLD, without an escalation in adverse events. Notably, these favorable outcomes were independent of weight management. Recent research (15) have demonstrated that an intermittent fasting regimen of 5:2 effectively prevents the onset of non-alcoholic steatohepatitis (NASH) and improves both established NASH and fibrosis without altering overall caloric consumption, furthermore, this research has also elucidated the cooperative roles of peroxisome proliferator-activated receptor alpha (PPARα) and glucocorticoid signaling-induced phosphoenolpyruvate carboxykinase 1 (PCK1) as crucial hepatic mediators in the fasting response. We propose that the 5:2 IF diet holds promise as a dietary regimen for lifestyle intervention among individuals with MAFLD.

Hepatic steatosis and fibrosis represent pivotal histological parameters due to their diagnostic and staging significance in disease pathology (16). Advanced fibrosis stands out as the foremost predictor of mortality in MAFLD (17). Studies have explored the efficacy of the 5:2 IF diet in MAFLD treatment, reporting reductions in steatosis and fibrosis (18–20), consistent with our findings. Our study corroborates these prior observations, demonstrating that both the 5:2 IF diet and daily calorie restriction can ameliorate liver steatosis and fibrosis, as well as decrease liver enzyme markers ALT and AST in patients with MAFLD-related metabolic fatty liver disease, even with a brief 12-week intervention period. Additionally, significant enhancements were noted in the 5:2 diet group, with more patients exhibiting F0-1 degrees of hepatic fibrosis and fewer patients hepatic steatosis ≥moderate after 12 weeks of treatment.

The intriguing aspect is the independence of the liver benefits derived from the 5:2 IF regimen on weight loss; rather, they may stem from metabolic transitions between fasting and feeding states (21, 22). This metabolic shift promotes the utilization of fatty acids and ketones as energy substrates over glucose. Ketones are recognized as potent signaling molecules involved in regulating numerous cellular pathways, thereby conferring resistance to stress and disease while enhancing organ function (23). Although the precise mechanism underlying the promotion of liver health by IF remains elusive (24), animal data suggests that the beneficial effects of IF on liver metabolism and inflammation persist irrespective of alterations in dietary intake or weight loss.

Extensive investigation has consistently underscored the favorable impact of IF regimens or daily calorie restriction 12 weeks on body weight, typically resulting in a reduction of approximately 2–5 kg and a decline in BMI (25–27), echoing these findings, our study observed a similar weight loss trend of approximately 2 kg in both the 5:2 IF diet group and the daily calorie restriction cohort, albeit without statistical significance in terms of body weight, BMI, body fat, body fat rate, and muscle mass between the two groups as both the two groups have the same effect on these indicators. Body weight and BMI were decreased following a 12-week intervention in both groups, but statistical significance was not achieved. However, noteworthy reductions in WC, WHR, and visceral fat grade were observed after the 12-week intervention in both IF regimens and daily calorie restriction compared to baseline, consistent with findings from previous studies (7, 8). These reductions hold clinical significance within the context of MAFLD and overall metabolic health, as abdominal (visceral) obesity, regardless of body weight or BMI, is widely acknowledged as a primary risk factor for metabolic syndrome. Additionally, we also observed more obvious decreased in WC (−7 cm) because we included patients with higher body weight.

In the context of patients afflicted with MAFLD, 5:2 IF diet appears to exert a degree of hepatoprotective influence, as evidenced by substantial declines in ALT levels, a specific marker of hepatic injury. We observed a notable reduction in ALT and AST levels by 40 and 15 U/L, respectively, following 12 weeks of either the 5:2 IF diet or daily calorie restriction. Correspondingly, Johari et al. (18) reported reductions in ALT and AST levels by 25 and 8 U/L, respectively, among MAFLD patients subjected to alternate-day calorie restriction for 8 weeks. The authors attributed this decline in liver enzymes to improvements in visceral adiposity or hepatic steatosis (18). Both in the 5:2 IF diet group and daily calorie restriction cohort, although there was a significant decrease in ALT and AST following 12 weeks treatment, there was no statistically significant difference between the two groups. Furthermore, our investigation revealed a more pronounced amelioration in steatosis and fibrosis scores with the 5:2 IF diet compared to daily calorie restriction.

We observed a lower HDL-C level and a higher LDL-C level with the 5:2 IF diet compared to daily calorie restriction. Previous clinical trials have shown short-term time-restricted eating had inconsistent effects on glycemic control, insulin sensitivity, and lipids in obese persons (25, 28). It is plausible that the extent of weight reduction induced by the 5:2 IF diet or daily calorie restriction did not reach a magnitude sufficient to elicit improvements in lipid profile markers such as cholesterol and triglycerides. Moro et al.’s investigation revealed no alteration in plasma lipids following 8-h time-restricted feeding (29). Emerging data indicates that achieving a weight loss exceeding 5% is requisite for ameliorating plasma lipid levels and glucoregulatory parameters (30). Low plasma HDL-C is a constituent of the atherogenic dyslipidemia profile (2), the underlying mechanism governing the divergent anti-dyslipidemic effects of various IF modalities in the context of MAFLD remain unclear. We hypothesize that IF protocols involving ≤24 h of fasting (e.g., Alternate-Day Fasting and Time-Restricted Feeding) may stimulate heightened hepatic synthesis of HDL as an initial physiological response to lipid and carbohydrate deprivation (31).

Type 2 diabetes represents a recognized risk factor for the severity of MAFLD (32). Among MAFLD patients, those undergoing Ramadan fasting exhibited reductions in fasting blood sugar (FBS), insulin levels, and HOMA-IR scores, contrasting with the non-fasting group (33). A meta-analysis encompassing 12 studies involving 545 participants indicated a significant reduction in FBS associated with IF diets compared to control regimens (34). A recent randomized controlled trial (35) examined the impacts of a 5:2 dietary regimen and an exercise intervention against standard lifestyle education on glycemic control and cardiometabolic health in adults with overweight/obesity and type 2 diabetes. The findings indicated that a medically supervised 5:2 energy-restricted diet might offer an alternative strategy for enhancing glycemic control. Conversely, while the exercise regimen was effective in improving body composition, it did not significantly enhance glycemic control. In our study, HbA1c, and HOMA-IR was significantly decreased after 12- week both by 5:2 IF diet and daily calorie restriction eating, however, lower FBS, fasting insulin and hs-CRP were noted in the daily calorie restriction eating. These neutral effects on FBS, fasting insulin and hs-CRP of 5:2 IF diet may be attributed to the normoglycemic status of many patients in our cohort.

Elevated levels of inflammatory biomarkers among individuals with MAFLD may influence the pathogenesis of cardiovascular disorders. Hs-CRP, synthesized by the liver under the influence of pro-inflammatory cytokines, serves as a recommended marker for assessing low-grade inflammation and screening cardiovascular disease risk (36). Aliasghari et al. (33) documented reduced hs-CRP levels subsequent to Ramadan fasting. In our investigation, participants subjected to daily calorie restriction exhibited diminished hs-CRP levels at the study’s conclusion compared to baseline, whereas no significant change was observed in the 5:2 IF diet group. The rationale behind the superior reduction in inflammatory response with daily calorie restriction eating warrants further elucidation.

Various IF strategies show promise in managing MAFLD. IF encompasses a spectrum of energy restriction methods, ranging from alternating eating and fasting periods to complete fasting or very low-energy intake (37). A prospective observational trial involving 697 participants, including those with and without type 2 diabetes, demonstrated the efficacy of periodic fasting in reducing NAFLD (19). In another controlled trial, 70 patients with NAFLD were assigned to intermittent calorie restriction, a low-carbohydrate diet, or general lifestyle advice for 3 months. Participants undergoing intermittent calorie restriction exhibited reductions in both body weight and liver steatosis compared to those following lifestyle advice (20).

Research has validated the effectiveness of FibroTouch (FT) as a diagnostic tool for liver fibrosis, showing diagnostic accuracy similar to that of FibroScan (FS) (38, 39). FS, a vibration-controlled transient elastography device, is utilized for assessing liver stiffness and diagnosing liver fibrosis and hepatic steatosis (39). FS has been extensively studied, widely accepted, and integrated into major clinical guidelines (40). In 2013, FT employs vibration-controlled transient elastography to LSM and attenuation parameter, offering diagnostic capabilities for liver fibrosis and hepatic steatosis comparable to those of FS (41).

Three subjects documented experiencing sensations of hunger and fatigue on fasting days, whereas five participants expressed various discomforts such as feeling cold, headaches, diminished energy levels, and sporadic dizziness within the group adhering to daily calorie restriction. As anticipated, individuals adhering to the 5:2 IF diet experienced sensations of hunger and fatigue during fasting periods, but few other unforeseen side effects. While intermittent fasting has not been associated with major adverse events, common complaints include sensations of coldness, headaches, lethargy, and sporadic dizziness (42). Although generally considered safe for most individuals, recommending intermittent fasting to patients with type 2 diabetes undergoing insulin or sulfonylureas therapy poses challenges due to the risk of hypoglycemia (42).

## Limitations

This study is subject to certain limitations, as are our experimental methodologies. Firstly, our investigation specifically targets the Asian demographic, employing short-term intermittent dietary restrictions over a span of 3 months. Future inquiries would benefit from extended durations (>12 weeks) or wider population cohorts, with clinical investigations aimed at discerning the significance of liver-related outcomes. Secondly, our study did not assess total energy expenditure. Additionally, physical activity remained uncontrolled as our primary objective was to examine the isolated effects of dietary regimens on weight reduction.

## Conclusion

In summary, although both 5:2 IF diet and daily caloric restriction eating achieved similar effect on body weight, liver enzymes, lipid profile and glycemic indices after 12 weeks treatment, 5:2 IF diet demonstrates better improvement in fibrosis and steatosis scores independently from weight regulation. Consequently, it is anticipated to emerge as a viable dietary modality for lifestyle intervention among patients diagnosed with MAFLD.

## Data Availability

The raw data supporting the conclusions of this article will be made available by the authors, without undue reservation.
