# Supplementary material for: Effect of 5:2 intermittent fasting diet versus daily calorie restriction eating on metabolic-associated fatty liver disease—a randomized controlled trial
Source: Front Nutr. 2024 Aug 20;11:1439473. doi: 10.3389/fnut.2024.1439473 (PMC11368853; doi:10.3389/fnut.2024.1439473)
Supplement: Supplementary file 1 [file Presentation_1.pdf]

1. Detail of 52 intermittent fasting diet versus daily calorie restriction eating program

(1) In February 2020, the international expert group reached an international consensus to replace the existing name "nonalcoholic fatty liver disease (NAFLD)" with "Metabolic associated fatty liver disease (MAFLD)"[1]. Fatty liver in individuals with overweight/obesity, type 2 diabetes or multiple metabolic disorders is defined as MAFLD[1].

Specifically, a patient has no history of excessive alcohol consumption (male alcohol consumption is less than 30 g/d, female alcohol consumption is less than 20 g/d). The imaging or histological diagnosis is diffuse hepatocellular degeneration. Excluding other factors such as genotype 3 hepatitis C infection, autoimmune liver disease, medication (tamoxifen, amiodarone, sodium valproate, methotrexate, glucocorticoids, etc.), Wilson's disease, total parenteral nutrition, inflammatory bowel disease, celiac disease, hypothyroidism, Cushing's syndrome, etc β Lipoprotein deficiency, lipoatrophic diabetes, Mauriac syndrome and other causes that can lead to fatty liver

(2) **Treatment plan for MAFLD in this hospital**

(a) Exercise intervention: Provide moderate intensity exercise intervention to all patients, and recommend brisk walking exercise for no less than 30 minutes per day and no less than 5 days per week.

(b) Drug intervention: ① MAFLD patients with elevated levels of AST and ALT should be treated with Yishanfu 2 #/time, three times a day oral administration of Ligaron tablets 1 #/time, three times a day oral administration; ②Patients with MAFLD and insulin resistance should receive metformin sustained-release tablets at a dose of 1.0/time, once a day orally; ③Patients with MAFLD combined with hypertriglyceridemia should receive 0.2g/dose of fenofibrate tablets orally once a day; ④MAFLD patients with hyperuricemia are given sodium bicarbonate tablets at a dose of 1.0g/time, orally administered three times a day, and non buxostat tablets at a dose of 40mg/time, orally administered once a day.

2. Measurement details for waist circumference (WC), hip circumference (HC), and waist-to-hip ratio (WHR) are outlined as follows:

(1) Waist Circumference (WC) is typically measured at the midpoint between the anterior superior iliac crest and the lower edge of the 12th rib, or at the level of the navel. The subject stands with feet together or separated by 25 to 30 centimeters, evenly distributes weight, relaxes the abdomen, and allows arms to naturally droop. The tape measure is placed at the selected point, ensuring a snug but not compressed fit parallel to the ground. Measurement is taken at the end of exhalation and before inhalation, noting the intersection of the 0 mark with the tape for the waist circumference size. Smooth breathing is maintained throughout, without holding breath or tightening the stomach. Measurements are conducted at least twice consecutively, with a maximum allowable difference of 1cm between readings, and the average is recorded as the final result.

(2) Hip Circumference (HC) measurement requires the subject to stand upright with natural relaxation, avoiding tightness or slouching. The tape measure is positioned horizontally over the pubic bone and the most prominent part of the gluteus maximus, ensuring a firm contact with the skin. The measurement is taken under natural breathing conditions.

(3) Waist-to-Hip Ratio (WHR) is calculated as the ratio of waist circumference to hip circumference (WHR = WC/HC).

References

[1] Eslam M, Sanyal AJ, George J, International Consensus Panel. MAFLD: A Consensus-Driven Proposed Nomenclature for Metabolic Associated Fatty Liver Disease. Gastroenterology. 2020. 158(7): 1999-2014.e1.
